# Clinical Pharmacology and Pharmacokinetics of Levetiracetam

**DOI:** 10.3389/fneur.2013.00192

**Published:** 2013-12-04

**Authors:** Chanin Wright, Jana Downing, Diana Mungall, Owais Khan, Amanda Williams, Ekokobe Fonkem, Darin Garrett, Jose Aceves, Batool Kirmani

**Affiliations:** ^1^Division of Pharmacy, Department of Pediatrics, Scott & White Hospital and Texas A&M Health Science Center College of Medicine, Temple, TX, USA; ^2^Texas A&M Health Science Center College of Medicine, Temple, TX, USA; ^3^Division of Neonatology, Department of Pediatrics, University of Chicago Medical Center, Chicago, IL, USA; ^4^Department of Neurology, Scott & White Neuroscience Institute and Texas A&M Health Science Center College of Medicine, Temple, TX, USA; ^5^Texas A&M University, Temple, TX, USA; ^6^Division of Pediatric Neurology, Department of Pediatrics, Scott & White Hospital and Texas A&M Health Science Center College of Medicine, Temple, TX, USA; ^7^Epilepsy Center, Department of Neurology, Scott & White Neuroscience Institute and Texas A&M Health Science Center College of Medicine, Temple, TX, USA

**Keywords:** levetiracetam, pharmacokinetics, epilepsy

## Abstract

Status epilepticus and acute repetitive seizures still pose a management challenge despite the recent advances in the field of epilepsy. Parenteral formulations of old anticonvulsants are still a cornerstone in acute seizure management and are approved by the FDA. Intravenous levetiracetam (IV LEV), a second generation anticonvulsant, is approved by the FDA as an adjunctive treatment in patients 16 years or older when oral administration is not available. Data have shown that it has a unique mechanism of action, linear pharmacokinetics and no known drug interactions with other anticonvulsants. In this paper, we will review the current literature about the pharmacology and pharmacokinetics of IV LEV and the safety profile of this new anticonvulsant in acute seizure management of both adults and children.

## Introduction

Intravenous levetiracetam (IV LEV) is a second generation antiepileptic currently approved by the FDA as an adjunctive treatment in patients 16 years of age and older as an anticonvulsant when oral therapy is not tolerated (1). The intravenous formulation was approved in 2006. The cornerstone of therapy remains the older intravenous antiepileptic drugs (AEDs) such as benzodiazepines, phenytoin, and phenobarbital, all of which have unwanted interactions and side effects. Increasing use in clinical practice for the management of acute seizures warrants a review of the pharmacology, pharmacokinetics, and safety profile of this drug.

## Pharmacology

Levetiracetam is an (*S*)-enantiomer of the ethyl analog of piracetam, in the class of nootropic drugs which are considered to be “pharmacologically safe” (2, 3). It is structurally unrelated to any other antiepileptic class and has a novel mechanism of action. Although the precise mechanism is unknown, in animal models it has been shown to bind to synaptic vesicle protein SV2A. This protein has been related to modulation of synaptic vesicle exocytosis and neurotransmitter release. Animal models show that the affinity for SV2A is associated with protection against seizures making it an important target for new AEDs (4). *In vitro* studies demonstrated oppositional activity to negative modulators of gamma-aminobutyric acid (GABA)-gated currents despite lack of binding affinity to GABA receptors (5, 6).

## International Licensing Indications for Levetiracetam

Levetiracetam is approved for partial seizures. The parenteral form is approved as an alternative for treatment of partial seizures if oral form is not feasible.

Dosage and directions: PO/IV-Adjunctive therapy for partial seizures – the initial dose is 500 mg twice daily with gradual upward titration to a maximum of 3 g/day.

The dose is the same for monotherapy and for partial seizures with and without secondary generation (1).

Minimum: 500 mg twice daily

Maximum: 3 g daily

## Pharmacokinetics

Levetiracetam is rapidly and almost completely absorbed (96%) after oral administration. Normal time to maximum concentration (*C*_max_) is delayed from 1 to 1.5 h when administered with food, but the extent of absorption is not affected. *C*_max_ is decreased by 20% as well. There is little protein binding (<10%), therefore it does not compete with other drugs for binding sites (7). The volume of distribution ranges from 0.5 to 0.7 l/kg in adults and 0.6–0.9 l/kg in premature infants and children (2, 8). Ramael and colleagues evaluated the single-dose bioavailability of an intravenous (IV) LEV relative to oral tablets and IV relative to placebo pharmacokinetics and multiple-dose tolerability in 18 healthy subjects. There were nine white females and nine white males. The first phase of the study was a single-dose, randomized, open-label, 2-way crossover comparison of bioavailability of a 15-min infusion of LEV 1,500 mg and three 500 mg oral tablets. After this, subjects entered the second phase, which was a multiple-dose, randomized, double-blind, placebo-controlled, parallel-group tolerability, and pharmacokinetic study. They received nine successive doses of LEV 1,500 mg IV or placebo every 12 h. Plasma LEV concentrations were measured and the researchers compared the bioavailabilities. The IV infusion and oral tablet had a similar *C*_max_ (50.5 and 47.7 μg/ml, respectively) and AUC (392.4 and 427.9 μg h/ml, respectively) after a single dose. The IV and oral formulations were bioequivalent, indicated by the findings that geometric mean IV/oral ratios were 92.2 (90% CI, 89.0–95.6) for AUC and 103.7 (90% CI, 91.6–117.4) for *C*_max_. Within 48 h steady state was reached after multiple twice-daily infusions. The incidence of treatment-emergent adverse events was 89% (16/18) for the IV formulation and 72% (13/18) for the oral tablets during the single-dose phase. During the multiple-dose phase the incidence of treatment-emergent adverse events was 67% (8/12) in the IV LEV group and 33% (2/6) in the placebo group. Somnolence (33 vs. 17% placebo) and postural dizziness (25 vs. 0% placebo) were the most common adverse events with IV LEV in the multiple-dose phase (9).

Ramael and colleagues also performed a phase I, randomized, single-blind placebo-controlled study to evaluate the safety and tolerability of LEV administered intravenously at higher doses and/or at a faster infusion rate than proposed. Forty-eight healthy subjects (three male and three female patients per dose) were randomized to receive IV infusion single-ascending doses of LEV, administered at different dosages vs. placebo (1,500, 2,000, 2,500 mg over 5 min; 2,000, 3,000, 4,000 mg over 15 min). Healthy subjects were aged 18–55 years of age, had a body mass index (BMI) of 19–28, had to be in good physical and mental health, and were excluded for any disorder that could alter the pharmacokinetics or be a risk factor to not tolerating the drug (e.g., allergies, previous intolerance of pyrrolidone derivatives or its injectable diluents).

Adverse events most commonly reported were dizziness (52.8%), somnolence (33.3%), fatigue (11.1%), and headache (8.3%). There was no clear relation with IV dose level or infusion rate, and were consistent with the oral formulation safety profile. Each dose level and for both IV infusion rates had similar safety profiles. The pharmacokinetics of LEV administered by IV infusion were comparable across dose groups and infusion rates. The geometric means for 4,000 mg administered over 15 min and 2,500 mg infused over 5 min were maximum plasma concentration, respectively 145 (24.6%) and 94.3 (36.2%). Area under the plasma concentration-time curves were 1,239 (19.2%) and 585 (9.6%) μg/h/ml, and terminal half-lives were 8.0 (14.5%) and 7.0 (12.7%) h. Thus, Ramael and colleagues surmised that the pharmacokinetic profile was consistent with oral LEV and that the higher rates than those proposed of LEV IV administration dosages and infusion rates were tolerated in healthy subjects (10).

In a crossover study conducted by Leppik and colleagues, five women and five men were given intramuscular (IM) and IV LEV. All subjects were healthy and ranged in age from 21 to 59 years old (mean 35.0 years). Subjects were randomized so that half of them first received the IM injection followed 2 weeks later with IV administration. The administration of IM LEV was double-blinded to fully assess tolerability. To determine absolute bioavailability, the IV administration was unblinded. IM LEV was determined to be well tolerated due to no observation of inflammation or tissue break down and the pain scores at 1 min after IM LEV (29 mm for women and 18 mm for men, both with significant subject variability) returning to a baseline 15 min after IM injection. Compared to IV, IM LEV was completely absorbed. The mean bioavailability was 1.08 ± 0.19 (0.94–1.12) with CI of 97–118%. Within 2 h after IM injection 85% of *C*_max_ was reached. Within 0.75–4 h (median = 2 h) of IM administration maximum concentration occurred. Two hour post-dose LEV concentrations were similar for both IV and IM doses. The study concluded that 5 ml (500 mg) IM LEV is well tolerated and its bioavailability is equivalent to an IV injection (11).

Wheless and colleagues evaluated the safety of a rapid loading dose of IV LEV in a prospective, open-label, single-center study conducted from February 2007 to August 2008. Patients had a confirmed diagnosis of generalized epilepsy or partial-onset seizures and received an AED prior to IV LEV. A total of 45 study patients, aged 4–32 years, were divided into three equal dosing groups of 20, 40, and 60 mg/kg. A single loading dose of IV LEV was administered using a flow control pump which aided in timing of infusion accuracy. The 20 and 40 mg/kg group doses were administered as a 5-min infusion, and the 60 mg/kg dose was administered as a 6-min infusion. Baseline hematology and serum chemistries were collected upon hospital admission. During the infusion, safety assessments, and electrocardiograms (EKGs) were performed. The serum LEV concentrations were 14–189 μg/ml after infusion. There were no EKG abnormalities, no local infusion site redness or tenderness, and no changes in blood pressure. The mean dose administered in the 5- and 6-min infusion groups were 26.1 and 51.3 mg/kg. The 5- and 6-min infusion groups had a comparable volume of distribution (l/kg), with a mean of 0.40 and 0.42, respectively. Within 15 min of the end of the infusion of IV LEV, 95% (38/40 patients) had achieved maximum plasma drug concentrations. The researchers concluded a rapid infusion can safely achieve high serum levels of parenteral LEV (12).

### Hepatic impairment

No dosage adjustment is needed for patients with hepatic impairment. However in patients with severe hepatic impairment, Child-Pugh C, total body clearance was half that of normal subjects. Decreased renal clearance was attributed for most of the decrease (1). In an open-label, parallel-group, single-dose pharmacokinetic study, Brockmoller and colleagues studied the pharmacokinetics of LEV and its metabolite UCB L057 in patients with liver cirrhosis. Five healthy subjects and patients with Child-Pugh class A (*n* = 5), B (*n* = 6), or C (*n* = 5) alcohol-induced cirrhosis received a single-dose of LEV. Biochemical liver function parameters were measured and correlated with the pharmacokinetics of LEV. Three dynamic liver function tests characterized liver function during the screening phase with the caffeine test, lidocaine test, and d-sorbitol as a probe for liver blood flow. After these tests were used to determine the baseline function, a 1,000 mg dose of LEV was administered to measure the pharmacokinetics of LEV and UCB L057. Validated gas chromatographic assays measured plasma and urine levels of LEV.

A deterioration of liver function was revealed by dynamic liver function tests. The healthy subjects and class A or B cirrhosis did not differ in their pharmacokinetics of LEV or UCB L057. A statistically significant 57% reduction was found in LEV total clearance in patients with class C cirrhosis (*p* < 0.001). The Child-Pugh class C vs. control of the geometric mean ratio of the area under the plasma concentration-time curve (AUC) for LEV was 2.41. The geometric mean of the half-life ratio was 2.27. The conclusion was that patients with mild to moderate liver impairment do not require a dose adjustment of LEV. However, half of the commonly recommended dose should be given initially to patients with severe cirrhosis (13).

### Renal impairment

The majority (66%) of LEV is eliminated renally as unchanged drug primarily by glomerular filtration with some subsequent tubular reabsorption (7). Mean half-life in infants and young children of 5.3 h is slightly shorter than the half-life in older children of 6 h. Adults have a reported half-life of 6–8 h and reach steady state concentrations in 2 days (7, 8). Total body clearance in infants <6 months is 1.23 ml/min/kg, >6 months is 1.57 ml/min/kg, and in adults approximately 1 ml/min/kg (9). Renal clearance in children is 0.8 ml/min/kg, 0.6 ml/min/kg in adults, and 0.5 ml/min/kg in the elderly (2). Renal failure can be expected to prolong the half-life to approximately 25 h (7). Doses in patients with altered renal function should be reduced (7). Nearly 50% of the drug can be removed by a 4 h dialysis session (1). The half-life of LEV was found to be 2.5 h longer in the elderly population most likely due to their decreased renal function (1).

### Pregnancy

The pharmacokinetic profile of AEDs is known to be affected by pregnancy, leading to concerns of seizure control, and fetal drug exposure. Tomson and colleagues studied the pharmacokinetics of LEV during pregnancy, delivery, lactation, and the neonatal period. In this prospective study, 14 women with epilepsy treated with LEV during pregnancy, and lactation during 15 pregnancies, were included to determine the LEV concentration in plasma and breast milk. The women’s ages ranged from 21 to 37 years old. During pregnancy, LEV was used as monotherapy in six patients and used as combination therapy in nine patients. Each trimester and after delivery, maternal plasma samples were collected. Maternal blood samples were collected at delivery, from the umbilical cord, and 2 days after delivery, blood samples were obtained from the newborns. Breast milk and plasma were collected from 11 mothers and their suckling infants after birth to determine LEV concentration.

The maternal/umbilical cord plasma concentration ratios ranged from 0.56 to 2.0 with a mean of 1.15. Neonatal plasma LEV concentration had an estimated half-life of 18 h. The mean milk/maternal plasma concentration ratio was 1.05 (range 0.78–1.55). The LEV dose in infants was estimated to 2.4 mg/kg/day. Breastfed newborn plasma concentrations were 13% of the maternal plasma levels. When compared with the baseline concentrations outside of pregnancy, during the third trimester maternal plasma concentrations were only 40% of baseline. There appears to be enhanced elimination of LEV in pregnancy. The resulting significant decline in plasma concentration indicates that therapeutic monitoring in pregnancy may be valuable (14).

### Children and neonates

Weinstock and colleagues assessed tolerability, safety, and pharmacokinetics of IV LEV in 52 children with epilepsy in a prospective, single-arm, multicenter study. Eligible children were aged 1 month to <4 years and 4–16 years with epilepsy requiring short-term in-hospital IV LEV administration. Children with difficult venous access, EKG abnormalities, ketogenic diet, felbamate exposure within 18 months, and status epilepticus in the previous 3 months were excluded. On study day 1, LEV pharmacokinetic assessments were performed from blood and saliva at 3–10 min intervals after the start of the infusion, at the end of the infusion, and up to 12 h post-infusion. The study completion rate of 16 of the 19 patients in the 1 month to <4 year group and 33/33 in the 4–16 years group. The seizures types in the 1 month to <4 year group vs. the 4–16 years group were partial onset 15/19 vs. 25/33, generalized onset 6/19 vs. 12/33, and unclassified 2/19 vs. 10/33. Sixty-three percent of patients had mild to moderate treatment-emergent adverse events. These were most frequently pyrexia and dry mouth. The LEV plasma and saliva concentration ranges were at expected levels based on the administered dose. The researchers concluded that IV LEV in the acute setting was overall well tolerated in children 1 month to 16 years (15).

Merhar and colleagues enrolled neonates in a prospective study to determine the pharmacokinetics of LEV. Eighteen neonates admitted to the neonatal intensive care unit were enrolled who were ≤30 days of age and ≥32 weeks gestational age with seizures treated with LEV from October 2008 to May 2010. Neonates with birth weights <2,000 g and creatinine levels ≥2.9 mg/dl were excluded. Before the first dose of LEV was administered, blood draws were taken. A dose of at least 20 mg/kg of phenobarbital was given to all subjects prior to receiving LEV. Fifty-four total measurements of LEV blood levels were obtained at time points during the entire dosing interval and concentrations were quantified by a liquid chromatography–electrospray tandem mass spectrometry assay. The pharmacokinetic analyses were performed with non-linear mixed effects modeling. The initial loading doses of LEV were between 14.4 and 39.9 mg/kg. The model prediction of the median maximum drug concentration was 39.8 mg/l (14.8–91.9 mg/l). One hour after a 30 mg/kg dose was the highest measured concentration at 87.6 mg/l.

When compared with older children and adults, neonates were found to have a lower clearance (neonates = 1.21 ml/min/kg, adults = 0.96 ml/min/kg), higher volume of distribution (neonates = 0.89 l/kg, adults = 0.5–0.7 l/kg), and longer half-life (neonates = 8.9 h, adults = 6–8 h). LEV was well tolerated in this population. The only adverse effect observed was mild somnolence 24 h after LEV administration. Thus, Merhar and colleagues concluded that the pharmacokinetics of LEV in neonates differed from children and adults (16).

Ng and colleagues assessed the safety of IV LEV in 30 children (average 6.3 years, 0.5–14.8 years) with seizures in a prospective study from July 2007 to October 2008. Enrollment criteria included hospitalized in patients treated for seizures who were LEV naïve or had not received LEV 3 days prior to administration. Exclusion criteria were unstable patients (including status epilepticus), prior LEV allergy, or patients >15 years old. Subjects received a single-dose of IV LEV 50 mg/kg, up to a maximal dose 2,500 mg, over 15 min. Ten minutes after the infusion, a blood level of LEV was drawn. Then the patients continued IV LEV or oral LEV as tolerated. Seizure types, duration, frequency, and seizure outcomes were evaluated via hospital chart review. The 50 mg/kg LEV dose was well tolerated by all patients and was a safe, appropriate loading dose. There were no observations of serious adverse reactions, although sleepiness, fatigue, and restlessness were noted. 10 min after the infusion a blood level of LEV was performed. LEV levels ranged from 47 to 128 μg/ml with a mean of 83.3 μg/ml. All seizure types had an apparent decrease in seizure frequency from 24 h before compared to 24 h after the infusion. Ng and colleagues reported 15.4% (4/26) had no seizures before or after the 24 h infusion. In addition, 57.5% (15/26) of patients who were having seizures within 24 h before the infusion became seizure free. The authors also reported that 38.5% (10/26) had more than 50% reduction in seizures (17).

Glauser and colleagues also conducted a multicenter, open label, single-dose pharmacokinetic study to assess LEV and its major metabolite L057 in infants and young children who were diagnosed with epilepsy. Thirteen subjects were enrolled in the study with the age range between 2.3 and 46.2 months. One patient was excluded because of a medical condition. The subjects received a dose of 20 mg/kg administered as 10% solution followed by evaluation for 24 h to assess the pharmacokinetics. The samples were collected predose and at 1, 2, 4, 9, 12, 16, and 24 h. The half-life of LEV was found to be 5.3 ± 1.3 h and clearance was 1.46 ± 0.42 ml/min/kg. No serious side-effects were reported. The authors concluded that the mean half-life of the drug was shorter and clearance was much more rapid as compared to previously reported adult data. The authors suggested that larger doses of the drug, which are corrected for body weight, should be administered to infants and young children (18).

### Drug interactions

Otoul and colleagues studied 187 children aged 4–16 years with epilepsy being treated with adjunctive LEV to determine whether plasma concentration of carbamazepine, valproic acid, topiramate, and lamotrigine were affected. There were 95 males and 92 females with 94 subjects randomized to receive adjunctive treatment with LEV and 93 subjects receiving placebo. Data from a randomized placebo-controlled phase III trial in children receiving concomitant AEDs and adjunctive LEV were used to perform these retrospective analyses. During an initial 4-week titration period, LEV was increased in 20 mg/kg/day increments to a target dose of 60 mg/kg/day. Then a 10-week period of treatment remaining at 60 mg/kg/day was evaluated. Study patients were evaluated if they had at least 2 weeks of a constant dose of LEV/placebo, unchanged AED dose for 2 weeks, and a maximum of two concomitant AEDs was allowed. Blood samples were taken at each study visit for trough AED levels and LEV levels, including two to three baseline period visits and five visits over the evaluation period.

At baseline and during LEV treatment the geometric mean concentrations were carbamazepine 8.4 μg/ml vs. 8.1 μg/ml (coefficient of variation, CV = 30%; *n* = 35), valproic acid 83.8 vs. 82.5 μg/ml (CV = 38%; *n* = 23), topiramate 7.3 vs. 7.2 μg/ml (CV = 82%; *n* = 28), and lamotrigine 8.2 vs. 7.7 μg/ml (CV = 62%; *n* = 22). The mean concentration ratios (LEV/baseline and their 90% confidence intervals for each AED were unaffected when combined with LEV administration. When LEV was compared to placebo, no differences were observed. The researchers concluded that in children with epilepsy LEV does not affect plasma concentrations of carbamazepine, valproic acid, topiramate, or lamotrigine (19).

Freitas-Lima and colleagues assessed whether LEV elimination was influenced by enzyme inducing antiepileptic drugs (EIAEDs). Study subjects were healthy besides being diagnosed with epilepsy and were aged 18–65 years. Patients were excluded if they were pregnant. The 15 subjects included in the EIAED group were stable for at least 1 month of treatment with carbamazepine, phenytoin, or phenobarbital alone or in combination. The 15 subjects in the control group were matched patients not receiving AEDs. Subjects on valproate or other drugs influencing drug metabolism were excluded. At baseline and at frequent intervals, serum and urine LEV levels were measured after a single oral 1,000 mg dose. High performance liquid chromatography (HPLC) determined plasma LEV concentrations. There were no reports of adverse effects related to LEV doses. When compared to controls, the EIAED group showed significantly lower AUC values, shorter half-life (*p* = 0.02) and a higher LEV oral clearance (*p* = 0.01), with respective magnitude differences of 21, 16, and 26%. The conclusion was that this interaction could have clinical significance for some patients even though the magnitude of the effect was relatively modest (20).

## Efficacy and Safety

Intravenous levetiracetam is an effective AED for seizure control. In an observational, multicenter retrospective study, LEV’s efficacy was found to be dependent on the timing of administration. Forty patients were included and in approximately half (57%) of the patients, IV LEV was effective in a mean time of 14 h. In 26 of the patients, IV LEV was used as add-on treatment with an efficacy of 46.1%. As early treatment (either pretreatment with benzodiazepines or nothing) in 14 of the patients, IV LEV showed an efficacy of 78.5% leading to the conclusion that it is more effective as a first line agent and that it is more difficult to treat refractory status epilepticus (21). The use of IV LEV in neonates resulted in favorable efficacy and tolerability as described by Abend and colleagues (22), Khan and colleagues (23), and Michaelides et al (24) in a variety of seizure etiologies. Furwentsches and colleagues (25) and Ramantani and colleagues (26) have also demonstrated LEV efficacy in prospective studies in which LEV was used as a first line treatment. Li et al (27) also demonstrated that LEV is a safe and effective treatment for infants and children in an observational, prospective study. Kirmani and colleagues (28), Goraya and colleagues (29), and Gallentine and colleagues (30) also showed the efficacy of LEV in acute seizure management in children. In another study, Khan and colleagues showed the efficacy of IV LEV in preterm neonate seizure management (31). The literature shows that a dose range from 10 to 70 mg/kg can be used effectively in children (17, 22–33). However, IV LEV is not approved for status epilepticus because no randomized larger multicenter trials were done to evaluate the efficacy in status epilepticus.

Levetiracetam can be used for both partial and generalized epilepsies but limitations in terms of seizure types have been published in the form of case reports and retrospective case series. LEV has been reported to be associated with aggravation of myoclonus in children and with adolescents with juvenile myoclonic epilepsies (34, 35). Caraballo and colleagues reported data which revealed LEV induced worsening of seizures during continuous spikes and waves during slow sleep in children with refractory epilepsies (36). Similar data about increased frequency of absence seizures with LEV have also been reported in the literature (37).

## Adverse Effects

The most common side-effects of LEV are neurobehavioral, including fatigue, nervousness, generalized weakness, irritability, agitation, emotional lability, depression, mood swings, vertigo, anxiety, unsteadiness, seizures, memory loss, confusion, increased reflexes, paresthesias, aggression, cognitive decline, and increased risk of suicide (1, 38). Other common side-effects include hypersensitivity reactions, infections, myalgias, rhinitis, and anorexia (1). Neurobehavioral side-effects are the main cause of discontinuing the medications in most instances (38). There are several case reports and case series which report acute onset of psychosis with the initiation of LEV (39–41). Increased risk of suicide has also been reported in patients on LEV therapy (42, 43).

## Take Away Points

1. Levetiracetam is a novel AED which is approved as adjunctive therapy for partial-onset seizures both in adults and children 1 month and older.
2. The metabolism of LEV has no effect on the cytochrome P450 enzyme system so it is favorable in terms of no drug–drug interactions.
3. No dose adjustment is needed in hepatic impairment but dose needs to be adjusted in patients with renal impaired.
4. The dose used in double-blind placebo-controlled trials is 1,000–3,000 mg/day. No tolerance was observed and efficacy was maintained in long term studies.
5. The drug seems to be well-tolerated in pregnancy and teratogenic potential is less than first generation antiepileptics. The anticonvulsant levels seem to decline toward the latter part of pregnancy requiring close monitoring of the drug levels.
6. The most common side-effects are somnolence, dizziness, and asthenia. The other reported side-effects are irritability, agitation, aggressive behavior, and anger.
7. The intravenous formulation is approved for patients 16 years or older if oral administration of the drug is not feasible. However, the off – label use in adults and children for acute seizure management yielded favorable results. Further studies are needed to prove the efficacy of this drug for acute seizure management.

## Conclusion

The literature shows that LEV has a novel mechanism of action and unique pharmacokinetic profile to be used as a desirable antiepileptic choice in an acute inpatient setting. Our conclusions, on the other hand, are based on existing data which include case reports, case series, retrospective studies, and some prospective trials. However, there are limitations in that there are neurobehavioral side-effects of the drug. We believe that there is a need for larger, prospective, multicenter, randomized double comparative blind trials in order to further clarify the role of this anticonvulsant in acute seizure management.

## Conflict of Interest Statement

The authors declare that the research was conducted in the absence of any commercial or financial relationships that could be construed as a potential conflict of interest.
